# NUCKS promotes cell proliferation and suppresses autophagy through the mTOR-Beclin1 pathway in gastric cancer

**DOI:** 10.1186/s13046-020-01696-7

**Published:** 2020-09-21

**Authors:** Erhu Zhao, Liying Feng, Longchang Bai, Hongjuan Cui

**Affiliations:** 1 State Key Laboratory of Silkworm Genome Biology, College of Biotechnology, Southwest University, No.2 Tiansheng Road, Beibei District, Chongqing, 400716 China; 2 Cancer Center, Reproductive Medicine Center, Medical Research Institute, Southwest University, Chongqing, 400716 China; 3 NHC Key Laboratory of Birth Defects and Reproductive Health (Chongqing Key Laboratory of Birth Defects and Reproductive Health, Chongqing Population and Family Planning Science and Technology Research Institute), Chongqing, 400020 China; 4 Engineering Research Center for Cancer Biomedical and Translational Medicine, Southwest University, Chongqing, 400716 China; 5 Engineering and Technology Research Center for Silk Biomaterials and Regenerative Medicine, Chongqing, 400715 China; 6 Westa College, Southwest University, Chongqing, 400716 China

**Keywords:** NUCKS, Autophagy, mTOR, Beclin1, Gastric cancer

## Abstract

**Background:**

Nuclear casein kinase and cyclin-dependent kinase substrate (NUCKS), a novel gene first reported in 2001, is a member of the high mobility group (HMG) family. Although very little is known regarding the biological roles of NUCKS, emerging clinical evidence suggests that the NUCKS protein can be used as a biomarker and therapeutic target in various human ailments, including several types of cancer.

**Methods:**

We first assessed the potential correlation between NUCKS expression and gastric cancer prognosis. Then functional experiments were conducted to evaluate the effects of NUCKS in cell proliferation, cell cycle, apoptosis and autophagy. Finally, the roles of NUCKS on gastric cancer were examined in vivo.

**Results:**

We found that NUCKS was overexpressed in gastric cancer patients with poor prognosis. Through manipulating NUCKS expression, it was observed to be positively associated with cell proliferation in vitro and in vivo. NUCKS knockdown could induce cell cycle arrest and apoptosis. Then further investigation indicated that NUCKS knockdown could also significantly induce a marked increase in autophagy though the mTOR-Beclin1 pathway, which could be was rescued by NUCKS restoration. Moreover, silencing Beclin1 in NUCKS knockdown cells or adding rapamycin in NUCKS-overexpressed cells also confirmed these results.

**Conclusions:**

Our findings revealed that NUCKS functions as an oncogene and an inhibitor of autophagy in gastric cancer. Thus, the downregulation or inhibition of NUCKS may be a potential therapeutic strategy for gastric cancer.

## Background

Gastric cancer is the second most common cancer in China and the third leading cause of cancer-related deaths worldwide [1, 2]. Although surgery is still the most effective treatment modality for patients with resectable tumors, due to the low rates of early detection and diagnosis, many patients with gastric cancer in China have very poor survival [3]. Therefore, there is a need to better elucidate the pathogenesis of gastric cancer by identifying useful biomarkers and novel targets for treatment.

Nuclear casein kinase and cyclin-dependent kinase substrate (NUCKS), a novel gene first reported in 2001 [4], is a member of the high mobility group (HMG) family [5]. NUCKS is widespread in vertebrates [6], and plays a significant role in modulating chromatin structure and regulating cellular events, such as replication, transcription, and chromatin condensation [7–10]. A recent report showed that NUCKS1 can regulate the messenger RNA expression of mechanistic target of rapamycin (mTOR) and Cyclins in mammary epithelial cells [11]. Furthermore, other studies have demonstrated that NUCKS is highly expressed in different cancers such as lung cancer [12], breast cancer [13, 14], hepatocellular carcinoma [15] and colorectal cancer [16], and promotes cell aggressiveness in pancreatic and gastric cancer [11, 17]. These findings suggest that NUCKS is a potential oncogene that may influence tumorigenesis. However, the oncogenic role and detailed mechanism of NUCKS remain poorly understood.

Autophagy is an evolutionarily conserved catabolic process that provides pro-survival advantages to cells under stressful conditions, such as nutrient inadequacy, hypoxia and microbial infection [18, 19]. The most famous regulator of autophagy is the mammalian target of rapamycin, a serine/threonine kinase that activated by metabolic stimuli, including amino acids, growth factors, and energy sufficiency [20–23]. Typically, the classic mTOR signaling pathway is considered to be crucial negative regulator for the formation of autophagosomes, where mTOR activation by nutrients and growth factors leads to inhibition of autophagy through the phosphorylation of multiple autophagy-related proteins that promote autophagy initiation and autophagosomes nucleation [24–26].

In the present study, we demonstrated that NUCKS plays an oncogenic role in gastric cancer via an mTOR-mediated signaling pathway. NUCKS knockdown significantly reduced cell proliferation and induced autophagy via mTOR-Beclin1 pathway activation, indicating that NUCKS may be a potential therapeutic target for gastric cancer treatment.

## Materials and methods

### Reagents and antibodies

The NUCKS antibody (12023–2-AP) was purchased from Proteintech (Wuhan, China). Polybrene (sc-134,220) was purchased from Santa Cruz Biotechnology (Shanghai, China). Antibodies against mammalian target of rapamycin (mTOR) (ab32028) and phosphor-S2448 mTOR (ab84400) were purchased from Abcam (Shanghai, China). The Cyclin Antibody Sampler Kit (no. 9869), apoptosis antibody (no. 5625, 9664, 3498), Phospho-p70 S6K antibody (no.9234) and Autophagy Antibody Sampler Kit (no.4445) were purchased from Cell Signaling Technology (Shanghai, China). The 3-[4,5-dimethylthiazol-2-yl]-2,5-diphenyltetrazolium bromide (MTT) (M5655), 5′-bromo-2-deoxyuridine (BrdU), and dimethyl sulfoxide (DMSO) (D5879) were obtained from Sigma-Aldrich. The tubulin antibody, 4′, 6-diamidino-2-phenylindole (DAPI) and 3, 3′-diaminobenzidine (DAB) were purchased from Beyotime (Shanghai, China). HRP goat anti-mouse and goat anti-rabbit antibodies were purchased from KPL, Inc. (Maryland, USA). Alexa Fluor 488 goat anti-rabbit IgG (H + L), Lipofectamine 2000, and puromycin (A1113803) were obtained from Life Technologies (Shanghai, China). The BrdU antibody (ab6326) was purchased from Abcam (Shanghai, China). All antibodies were diluted according to the instructions. Chloroquine and rapamycin were acquired from Must Bio-technology (Chengdu, China) and Beyotime (Shanghai, China), respectively.

### Cell culture

All gastric cancer cell lines were purchased from ATCC (Rockville, MD, USA) and cultured in Roswell Park Memorial Institute-1640 medium supplemented with 10% FBS and 1% P/S. The 293FT cells were cultured in 293FT growth medium consisting of DMEM with 10% FBS, 1% P/S, 0.5 mg/mL G418, 4 mM L-glutamine, 0.1 mM nonessential amino acids, and 1 mM sodium pyruvate. The 293FT transfection medium, 293FT growth medium without P/S, and G418 were used in the present study. All cell lines were cultured at 37 °C under a humidified atmosphere with 5% CO_2_. Growth media, FBS, antibiotics, and supplements were acquired from Thermo Fisher Scientific (Chengdu, China).

### Transfection and infection

The negative control plasmid pLKO.1-shGFP was purchased from Addgene (Massachusetts, USA). The shNUCKS sequences (Table S1) were inserted into the pLKO.1 vector. As described previously, the cDNA sequence of human NUCKS was purchased from Youbio Biological Technology Co., Ltd. (Changsha, China), which was cloned into the pCDH-CMV-MCS-EF1-GFP-Puro vector provided by GeneCreate (Wuhan, China) [27]. The Beclin1 shRNA plasmid was purchased from Sigma-Aldrich (TRCN0000299864), and the plasmid GFP-LC3B [28] was a gift from Prof. Ning Gao at the Third Military Medical University, China. Lentiviruses were generated by co-transfecting the 293FT cell line with the packaging plasmids pLP1, pLP2, and pLP/VSVG and the corresponding shRNA plasmids. Lipofectamine 2000 was used for all transfections according to the manufacturer’s instructions. Virus-containing supernatants were harvested and used for cell infections at a final concentration of 4 mg/ mL polybrene. At 24 h after the final round of infection, the cells were cultured in the presence of 2 mg/mL puromycin, and the drug-resistant cells were selected and pooled for subsequent experiments.

### Patient data analysis

Patient and gene expression data were downloaded from the TCGA: The Cancer Genome Atlas (https://cancergenome.nih.gov) and UCSC Xena (https://xena.ucsc.edu/public/). Kaplan-Meier survival analysis was conducted based on the high versus low NUCKS expression cutoff determined using the R packages.

### Gene set enrichment analysis (GSEA)

To gain insight into the biological processes and signaling pathways associated with NUCKS expression in gastric cancer, GSEA was performed using the Broad Institute GSEA version 4.0 software. The TCGA database was downloaded from UCSC Xena (https://xena.ucsc.edu/public/). The gene sets used for the enrichment analysis were downloaded from the Molecular Signatures Database (MsigDB, http://software.broadinstitute.org/gsea/index.jsp).

### Quantitative and reverse transcriptional PCR

Total RNA was extracted from cells using TRIzol reagent (Invitrogen) according to the manufacturer’s protocol. Then, 2 μg of RNA was reverse transcribed into cDNA for each sample. The mRNA expression was based on Ct values and was normalized to the values of glyceraldehyde-3-phosphate dehydrogenase as a control. The relevant primer sequences are presented in Supplementary Table S2.

### Western blot analysis

Cells were lysed in lysis buffer containing 50 mM Tris-HCl (pH 7.5), 150 mM NaCl, 1% Nonidet P-40, 0.25% sodium deoxycholate, 0.1% SDS with a complete protease inhibitor cocktail (Roche) and phosphatase inhibitors (Sigma-Aldrich). The proteins in lysates were separated by sodium dodecyl sulfate–polyacrylamide gel electrophoresis and then transferred to polyvinylidene difluoride membrane. SDS-PAGE gels were calibrated using Magic Mark XP Western Standard (Invitrogen). After being blocked with skim milk for 2 h, the membrane sections were incubated with the primary antibody and the corresponding secondary antibody. Primary antibodies were used at a dilution according to the instructions, while secondary antibodies were used at a dilution of 1:5000. Finally, bound antibodies were detected by chemiluminescence using the ECL Prime Western blotting detection system (GE Healthcare), and images were analyzed with a Lumino Imager (LAS- 4000 mini; Fuji Film Inc.).

### Tissue assay

The human paraffin embedded stomach tissue array (ST812) was purchased from US Biomax (Rockville, MD) though Shanxi Alenabio Biotechnology ltd. (Shanxi, China). As the manufacturer’s suggested protocols, the array was incubated with NUCKS antibodies at 4 °C overnight, followed by incubation with HRP-conjugated secondary antibodies at room temperature for 3 h. Staining was visualized using DAB, and tissues were counterstained with hematoxylin. Information about age, gender and tumor stages of donors is available on the website https://www.biomax.us. The ethics for collecting human tissue samples and the informed consent of patients have been verified by US Biomax, lnc.

### Cell viability and proliferation assays

Cell growth curves were obtained using the MTT assay. To assess cell viability, cells were seeded at a density of 8 × 10^2^/per well in 200 mL of medium in 96-well plates. The MTT assay was performed at the indicated time points from day 0 to 5, according to the manufacturer’s instructions. All experiments were independently repeated three times.

For BrdU staining, 1 × 10^4^ cells were cultured on coverslips in 24-well plates. Then, the cells were incubated with 10 μg/ml BrdU for 30 min, washed with 1 × PBS and fixed with 4% paraformaldehyde for 15 min. Then, samples were blocked with 5% goat serum for 2 h before being incubated with a primary antibody against BrdU for 1 h, followed by Alexa Fluor® 594 secondary antibody. DAPI was used for nuclear staining, and the percentage of BrdU incorporation was calculated from at least 10 randomly selected fields.

### Flow Cytometric analysis

For cell cycle analysis, 1 × 10^6^ cells were harvested into 60 mm plates and fixed in ice-cold 70% ethanol overnight at 4 °C, incubated with propidium iodide, and then analyzed using flow cytometry (BD Biosciences, San Jose, CA, USA) with Cell Quest (BD Biosciences, San Jose, CA, USA).

### In vitro and in vivo tumorigenic assays

For the in vitro soft agar assays, growth medium with 0.6% agarose was added to 6-well plates to form the lower layer, while growth medium with 0.3% agarose and 7 × 10^2^ cells was added to form the upper layer. Colonies were imaged and recorded after 2–3 weeks of growth. Subcutaneous xenograft were used for the in vivo study. The lateral backside of NOD/SCID female mice was subcutaneously injected with 2 ~ 3 × 10^6^ cells. Cells in which NUCKS was stably silenced were implanted into the right side of each mouse, while control cells were implanted into the left side. Calipers were used to measure the tumor volume, which was calculated using the formula volume = (π/6) × length × width^2^. After 4 weeks of growth, the mice were euthanized, and tumors were removed and weighed. At the endpoint, the original tumors were imaged, and fixed in neutral buffered formalin, and embedded in paraffin. Hematoxylin and eosin (H&E) staining and immunohistochemical analysis were performed for histopathological evaluations of the tissues. All mice were raised and monitored under SPF conditions. Animal welfare and experimental procedures were carried out in accordance with the Guide for the Care and Use of Laboratory Animals and approved by the Animal Ethics Committee of Southwest University.

### Immunofluorescence staining assay

For immunofluorescence staining, 2 × 10^4^ cells were seeded on coverslips in 24-well plates and cultured for 24 h. The cells were then washed with PBS, fixed in 4% paraformaldehyde for 15 min at room temperature, and then permeabilized using 0.1% Triton X-100 for 10 min. Coverslips were blocked in 10% goat serum for 1 h, after which the cells were incubated with primary antibodies against microtubule-associated protein light chain 3 beta (LC3B) before being incubated with Alexa Fluor 488 goat anti-rabbit IgG (H + L) as the secondary antibody. Finally, DAPI was used for nuclear staining.

### Statistical analysis

All experiments were carried out in triplicate according to statistical parameters including sample size and significance analysis, as indicated in the figures. A two-tailed Student’s test was performed to calculate significance. Normally distributed data are expressed as the 95% confidence level range, whereas similar quantitative data are expressed as the means ± SD and *P* < 0.05 values were considered statistically significant.

## Results

### High NUCKS expression correlates with poor patient prognosis in human gastric cancer

**Fig. 1:**
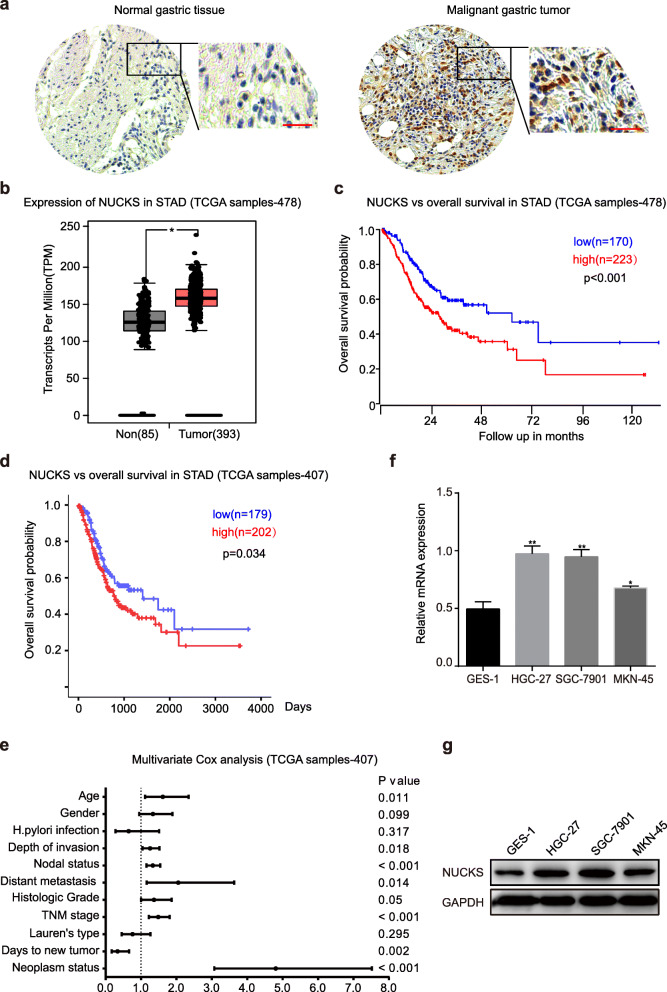
High expression of NUCKS correlates with poor patient prognosis in human gastric cancer. **a** The immunohistochemical assays of NUCKS expression in human gastric cancer tumors and adjacent normal tissue (Scale bars, 50 μm). **b** Box plot of NUCKS expression levels in peritumoral tissues (Normal) and gastric cancer tumors set with the log-rank test *P*-values < 0.05. **c** Kaplan-Meier analysis of progression-free survival and the log-rank test *P* values are indicated for the TCGA dataset (TCGA samples-478). **d** Kaplan-Meier analysis of progression-free survival and the log-rank test *P* values are indicated for the TCGA dataset (TCGA samples-407). **e** Multivariate cox regression analysis of independent predictors of the overall survival of patients with gastric cancer. **f**, **g** The qRT-PCR and Western blot assay were performed to detect NUCKS expression in gastric cancer cell lines

To investigate whether NUCKS is a novel biomarker for diagnosing gastric cancer, we first performed immunohistochemical staining on primary tissue microarray samples from gastric cancer patients. The results showed that NUCKS expression levels were significantly increased in the malignant gastric tumor compared to those observed in adjacent normal tissues (Fig. 1a). Then we confirmed the status of NUCKS as a putative amplified target in gastric cancer by evaluating data from the Cancer Genome Atlas (TCGA) and observing a significant difference in NUCKS levels in the tumors and adjacent normal tissues (*p* < 0.05; Fig. 1b). A Kaplan-Meier analysis of overall patient survival was performed to show that NUCKS was highly expressed in 223 of 393 cases of gastric cancer, and high expression was significantly correlated with reduced patient survival in the TCGA dataset (TCGA samples-478), whereas low NUCKS expression was correlated with good overall survival (*p* < 0.01; Fig. 1c). Similarly, high NUCKS expression was shown to be a factor for poor patient prognosis in datasets from other clinical databases, such as TCGA samples (407; Fig. 1d), TCGA samples (450; Fig. S1A), OncoLnc STAD samples (378; Fig. S1B). In addition, univariate cox regression analysis results showed that age, depth of invasion, lymph node metastasis, Lauren’s histological type and NUCKS expression were significantly interrelated with overall survival (Table 1). Furthermore, multivariate cox regression analysis confirmed age (*P* = 0.011), depth of invasion (*P* = 0.018), and lymph node metastasis (*P* < 0.001) as independent prognostic factors of the overall survival of patients with gastric cancer (Fig. 1e). Subsequently, we examined NUCKS expression in several gastric cancer cell lines (HGC-27, SGC-7901, and MKN-45) and normal stomach cells (GES-1) and observed that NUCKS was commonly expressed in all cell lines through both quantitative reverse transcription PCR assays and Western blot analyses. The cell line GES-1, which is a normal stomach cell line, exhibited relatively lower levels of NUCKS expression (Fig. 1f and g). Taken together, these results demonstrated that NUCKS could be a meaningful prognostic marker and may play an oncogenic role in tumor development.

**Table 1:** Correlation of NUCKS expression with Clinicopathological variables in TCGA data sets

Clinicopathological features		Cases	NUCKS expression				*F*	*P*
			Low	%	High	%		
Age	< 60	121	47	38.8	74	61.2	8.520	0.004
	≥60	286	156	54.5	130	45.5		
Gender	Male	144	77	53.5	67	46.5	1.150	0.284
	Female	263	126	47.9	137	52.1		
*H. pylori* infection	Negative	150	65	43.3	85	56.7	0.586	0.445
	Positive	19	10	52.6	9	47.4		
Depth of invasion	T1	22	13	59.1	9	40.9	4.210	0.041
	T2	69	40	58.0	29	42.0		
	T3	181	86	47.5	95	52.5		
	T4	33	17	51.5	16	48.5		
	T4a	48	20	41.7	28	58.3		
	T4b	24	10	41.7	14	58.3		
Lymph node metastasis	N0	123	66	53.7	57	46.3	6.040	0.014
	N1	108	63	58.3	45	41.7		
	N2	83	40	48.2	43	51.8		
	N3	74	27	36.5	47	63.5		
Distant metastasis	M0	358	179	50.0	179	50.0	1.687	0.195
	M1	27	17	63.0	10	37.0		
Histologic Grade	G1	10	4	40.0	6	60.0	0.534	0.462
	G2	150	81	54.0	69	46.0		
	G3	238	114	47.9	124	52.1		
Grade	Stage I	59	33	55.9	26	44.1	0.526	0.469
	Stage II	126	68	54.0	58	46.0		
	Stage III	156	63	40.4	93	59.6		
	Stage IV	42	27	64.3	15	35.7		
Lauren’s histological type	Intestinal type	82	49	59.8	33	40.2	4.475	0.036
	Diffuse type	66	28	42.4	38	57.6		
Days to new tumor event after initial treatment	< 326	31	15	48.4	16	51.6	0.063	0.803
	≥326	31	14	45.2	17	54.8		
Neoplasm status	Tumor free	185	102	55.1	83	44.9	3.005	0.084
	With tumor	74	32	43.2	42	56.8		

### NUCKS silencing reduces cell proliferation and regulates cell-cycle progression of gastric cancer cells

**Fig. 2:**
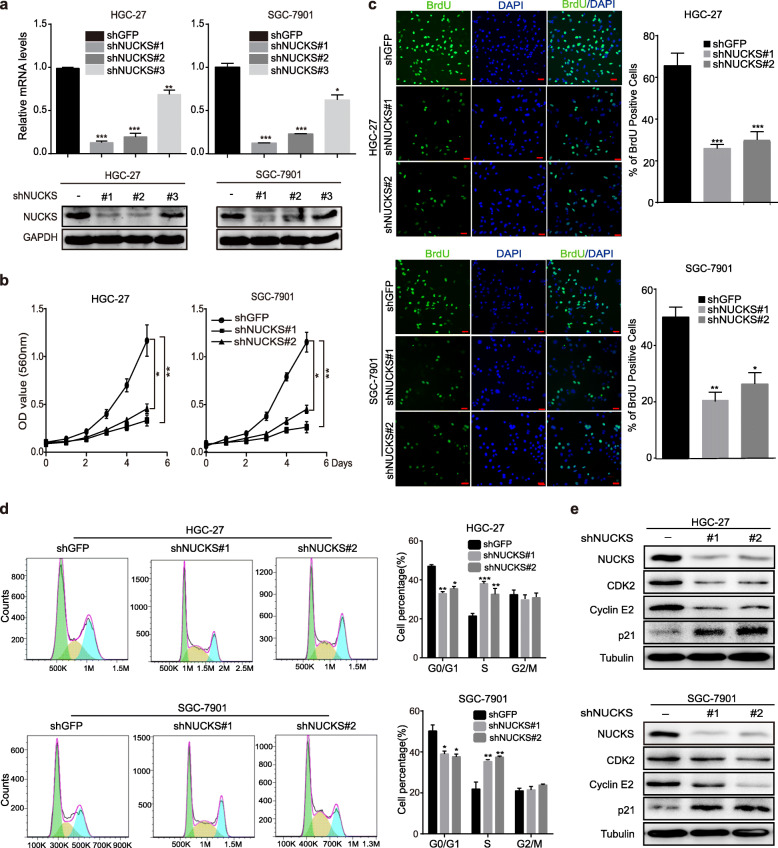
NUCKS silencing reduces cell proliferation and regulates cell-cycle progression of gastric cancer cells. **a** After NUCKS knockdown by shRNA in gastric cancer cell lines, NUCKS expression was detected using qRT-PCR and Western blot analysis. **b** NUCKS knockdown inhibited the proliferation of HGC-27 and SGC-7901 cells. MTT assay was performed to examine the effect of NUCKS knockdown on cell viability. **c** BrdU assays were performed after NUCKS knockdown. Representative images show immunofluorescence and the quantification of BrdU-positive cells (Scale bars, 20 μm). Data were analyzed using 2-tailed Student t tests (**P < 0.01, ***P < 0.001). **d** We analyzed the cell cycle distribution of HGC-27 and SGC-7901 cells by flow cytometry. **e** Western blot assay was performed to detect the expression of S cell cycle regulatory proteins in NUCKS-knockdown cells

Next, we knocked down NUCKS in two gastric cancer cell lines, HGC-27 and SGC-7901, by independently transducing three short hairpin RNA (shRNA) sequences, shNUCKS#1, #2 and #3. Western blot and qRT–PCR assay results showed that shNUCKS#1 and #2 most successfully knocked down NUCKS expression, whereas shNUCKS#3 exhibited a relatively lower efficiency in both HGC-27 and SGC-7901 (Fig. 2a). We then investigated cell viability after knocking down NUCKS in the two cell lines using shNUCKS#1 and #2 respectively. MTT assay results demonstrated that the shNUCKS groups resulted in a significant decrease cell growth (Fig. 2b). The 5′-bromo-2-deoxyuridine (BrdU) assay results consistently showed that the BrdU-positive rates in shNUCKS groups were much lower than those observed in the corresponding control groups (Fig. 2c). Then, we examined the cell cycle distribution of NUCKS knockdown and control cells by flow cytometry and observed that NUCKS knockdown induced cell-cycle arrest at S phase (Fig. 2d). To confirm the results, we measured the expression of some cyclins and CDKs, which can promote cells to pass the S-phase checkpoints and observed that the levels of CDK2, Cyclin E2 expression were decreased but that of p21 was increased following NUCKS knockdown (Fig. 2e). Taken together, these results indicated that NUCKS silencing can reduce cell proliferation and induce the cell-cycle arrest of gastric cancer cells.

### NUCKS restoration rescues the cell proliferation and cell-cycle arrest of NUCKS-silenced gastric cancer cells

**Fig. 3:**
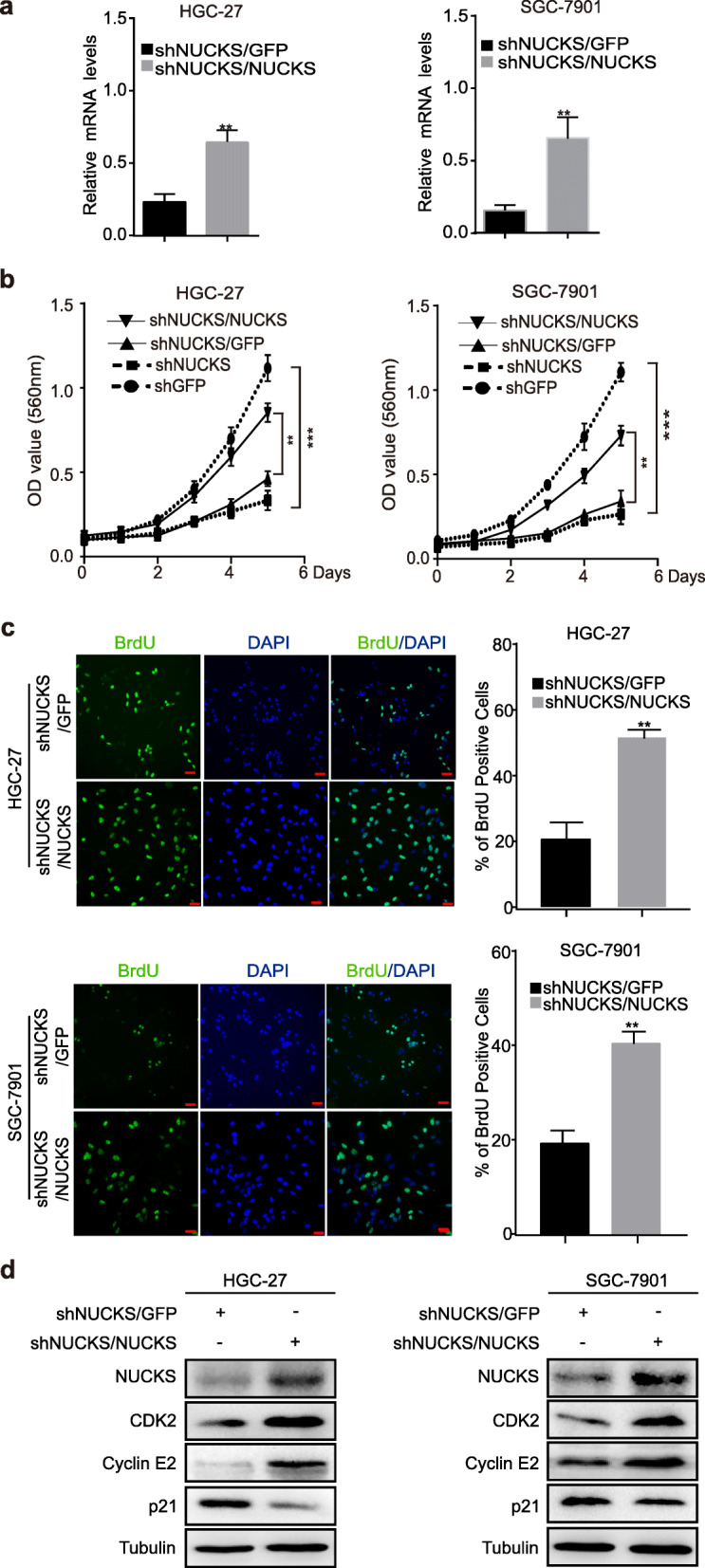
NUCKS restoration rescues the cell proliferation and cell-cycle arrest of NUCKS-silenced gastric cancer cells. **a** When NUCKS overexpressed in shNUCKS#1 knockdown cells, NUCKS expression was detected using qRT-PCR. The NUCKS-silenced cells were manufactured using the shNUCKS#1 plasmid in all follow-up experiments. **b** NUCKS restoration rescued the proliferation of HGC-27 and SGC-7901 cells. MTT assay was performed to examine the effect of NUCKS overexpressed on cell viability. **c** BrdU assays were performed after NUCKS overexpressed. Representative images show immunofluorescence and the quantification of BrdU-positive cells (Scale bars, 20 μm). Data were analyzed using 2-tailed Student t tests (**P < 0.01, ***P < 0.001). **d** Western blot assay was performed to detect the expression of S-phase cell cycle regulatory proteins in NUCKS-overexpressed cells

To further confirm the effect of NUCKS on cell proliferation, we overexpressed NUCKS in shNUCKS#1 knockdown cells. The overexpression efficiency was examined at the mRNA level, and the results showed that NUCKS was successfully overexpressed after being knocked down by shNUCKS#1(Fig. 3a). To investigate the influence of NUCKS overexpression on cell growth and proliferation, MTT and BrdU staining assays were performed. The results demonstrated that the restoration of NUCKS after knocking it down rescued cell growth and proliferation (Fig. 3b and c). Furthermore, we measured the expression of CDK2 and Cyclin E2, which can promote cells to go through the G1/S checkpoint by Western blot analysis. The data showed that the restoration of NUCKS rescued CDK2, Cyclin E2 and p21 expression (Fig. 3d). Taken together, these findings suggest that NUCKS is indispensable for gastric cancer cells growth and proliferation, and regulates cell cycle progression by downregulating the CCNE-CDK2 complex.

### NUCKS silencing induces autophagy and apoptosis in gastric cancer cells

**Fig. 4:**
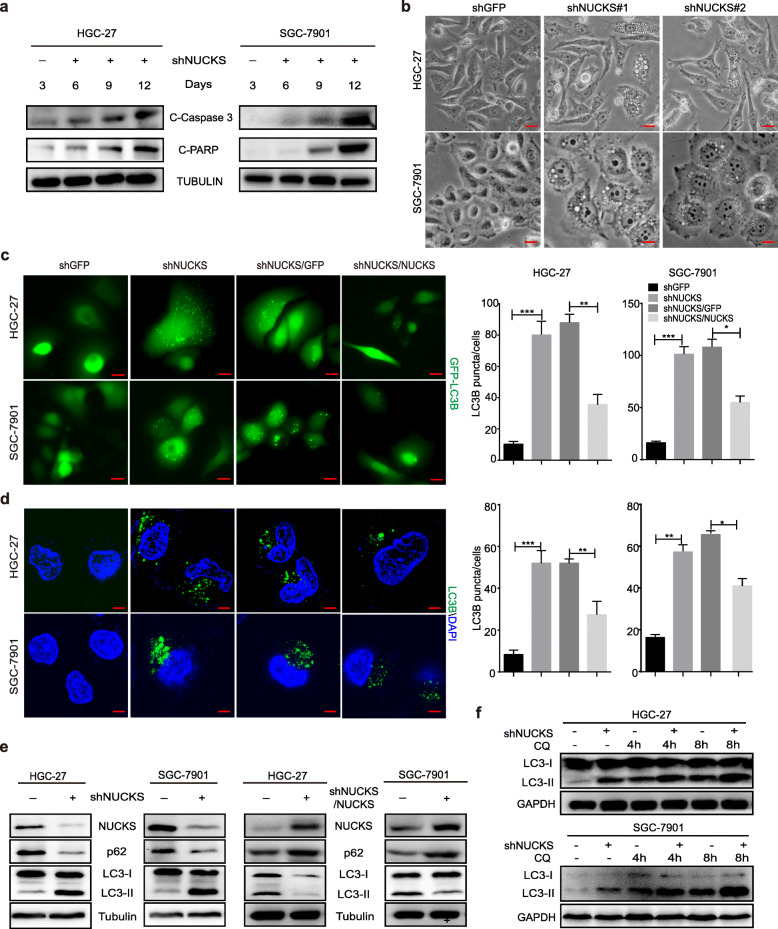
NUCKS silencing induces autophagy and apoptosis in gastric cancer cells. **a** Cleaved Caspase 3 and Cleaved PARP were detected by Western blot analysis after the downregulation of NUCKS. The drug-resistant cells were selected and continued to culture for 3, 6 and 9 days for Western blot analysis. **b** Detecting the change of gastric cancer cells morphology by light microscopy. **c** Cells transfected with the GFP-LC3B plasmid after NUCKS knockdown and restoration were examined using fluorescence microscopy (Scale bars, 10 μm). The quantification of LC3B-positive puncta is presented as a histogram (*P < 0.05, **P < 0.01, ***P < 0.001). **d** Immunofluorescence staining with a LC3B antibody was performed to confirm the induction of autophagy after NUCKS downregulation and upregulation. Representative LC3B-positive cells are shown. **e** The level of autophagy was evaluated by p62 and LC3B expression, as determined using Western blot analysis after NUCKS knockdown and restoration. **f** CQ blocked shNUCKS-induced autophagy. Cells were pretreated with 50 μM CQ and analyzed at indicated time points by Western blot

To elucidate the molecular mechanisms that underlie the observed NUCKS silencing-mediate inhibition on cell growth and proliferation, we first assessed whether NUCKS silencing could induce gastric cancer cell apoptosis by Western blot analyses. Western blot results showed the apoptosis proteins activated in gastric cancer cells after down-regulating NUCKS (Fig. 4a). This results are consistent with the reported role of NUCKS in the induction of apoptosis in gastric cancer cells [29]. The light microscope images showed that cells changed morphology and displayed high number of vacuoles after silencing NUCKS (Fig. 4b). Therefore, further experiments were performed to investigate the effect of NUCKS on autophagy status in gastric cancer cells. As LC3B is a specific marker of autophagy initiation, we transiently transfected gastric cancer cells with a GFP-LC3B plasmid and examined the cells under a fluorescence microscope. Interestingly, the shNUCKS#1 group showed a punctate pattern of GFP-LC3B fluorescence that indicated the recruitment of LC3B-II to autophagosomes and the formation of autophagic vacuoles. This result differed from the diffuse LC3B-associated green fluorescence observed in the control group. In contrast, in NUCKS-restored gastric cancer cells, this punctate pattern of GFP-LC3B fluorescence was significantly decreased compared to that observed in the shNUCKS#1/GFP group (Fig. 4c). To further confirm our findings, immunofluorescence analysis was performed to assess the conversion of LC3B-I to LC3B-II and the progression of autophagy. As predicted, the formation of LC3B-positive puncta markedly increased after NUCKS silencing and decreased after NUCKS rescue (Fig. 4d). These results are in agreement with the biochemical findings, as demonstrated by the increased conversion of LC3B and decreased levels of p62 (Fig. 4e). To distinguish whether the increase in LC3-II was due to autophagy induction or a block in downstream steps, LC3 turnover assays were performed. As shown in Fig. 4f, using CQ, an inhibitor of autophagic flow, we demonstrated that autophagy was induced by NUCKS knockdown. Taken together, our results demonstrated that NUCKS silencing induces autophagy in gastric cancer cells.

### NUCKS silencing induces gastric cancer cell autophagy via the mTOR-Beclin1 signaling pathway

**Fig. 5:**
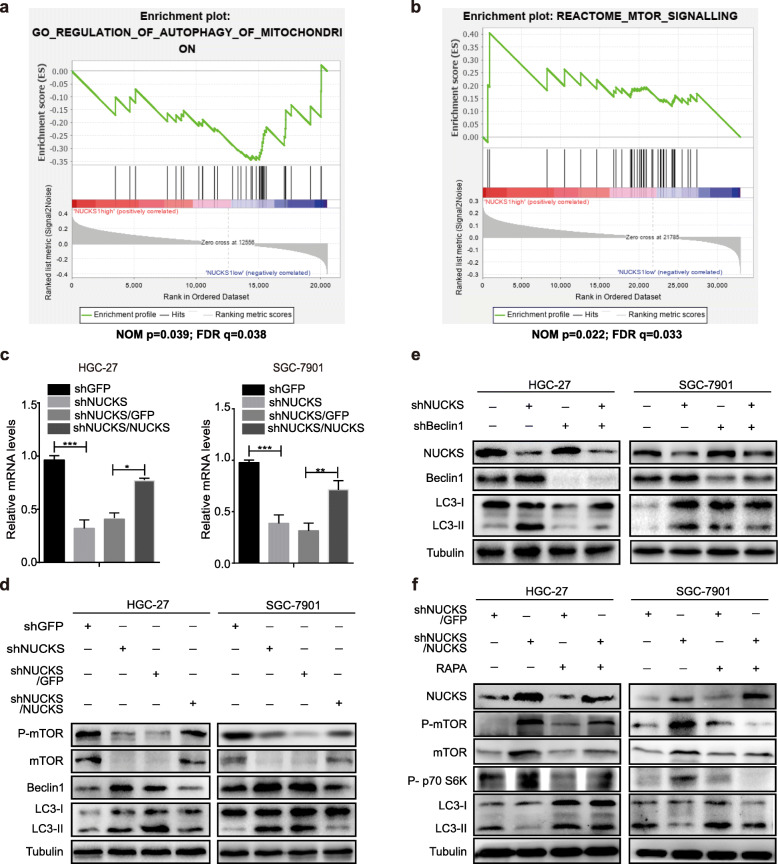
NUCKS silencing induces gastric cancer cell autophagy via the mTOR-Beclin1 signaling pathway. **a**, **b** GSEA shows significant enrichment of gene sets involved in autophagy process (left) and mTOR signal pathway (right). NOM = nominal, FDR = false discovery rate. **c** The quantitative analysis of mTOR expression in after NUCKS knockdown and restoration (**P < 0.01, ***P < 0.001). **d** The protein expression of mTOR pathway in NUCKS-knockdown and overexpression cells were measured by western blot assay. **e** Western blot analysis of mTOR, Beclin1 and LC3B expression when silencing Beclin1 on NUCKS-knockdown cells. **f** Western blot analysis of the mTOR signaling pathway after 10 nM Rapamycin treatment in NUCKS-restored cells

To investigate the role of NUCKS in gastric cancer progression, GSEA analyses were used to evaluate the potential pathways through which NUCKS functions. We observed that NUCKS was negatively associated with the autophagy process (Fig. 5a), and positively associated with the mTOR signaling pathway (Fig. 5b). To test whether downregulation of NUCKS silencing induces autophagy through the mTOR pathway, the mTOR and downstream protein expression was assessed by Western blot analysis. As shown in Fig. 5c, NUCKS knockdown obviously upregulated Beclin1 levels and downregulated those of phosphorylated mTOR (Ser-2448) and total mTOR level compared to that observed in the control group. In contrast, the levels of these proteins in the NUCKS-restored groups were all significantly rescued compared with that observed their corresponding control groups (Fig. 5c), and the downregulation of mTOR levels was also confirmed by qRT–PCR assays (Fig. 5d). To further to confirm the above results, we knocked down Beclin1 in NUCKS-silenced HGC-27 and SGC-7901 cells and observed that the recruitment of LC3B-II was also blocked (Fig. 5e). Then, we performed a rescue experiment and inhibited the mTOR pathway using rapamycin, a mTOR inhibitor. Compared with that observed in the control groups, rapamycin treatment partly reversed the effect of NUCKS overexpression on the rescue of proteins in the mTOR signaling pathway (Fig. 5f). Taken together, these data suggest that autophagy activation resulting from NUCKS silencing is mainly dependent on mTOR-Beclin1 pathway activation.

### NUCKS promotes colony formation in vitro and gastric cancer cell tumor formation in vivo

**Fig. 6:**
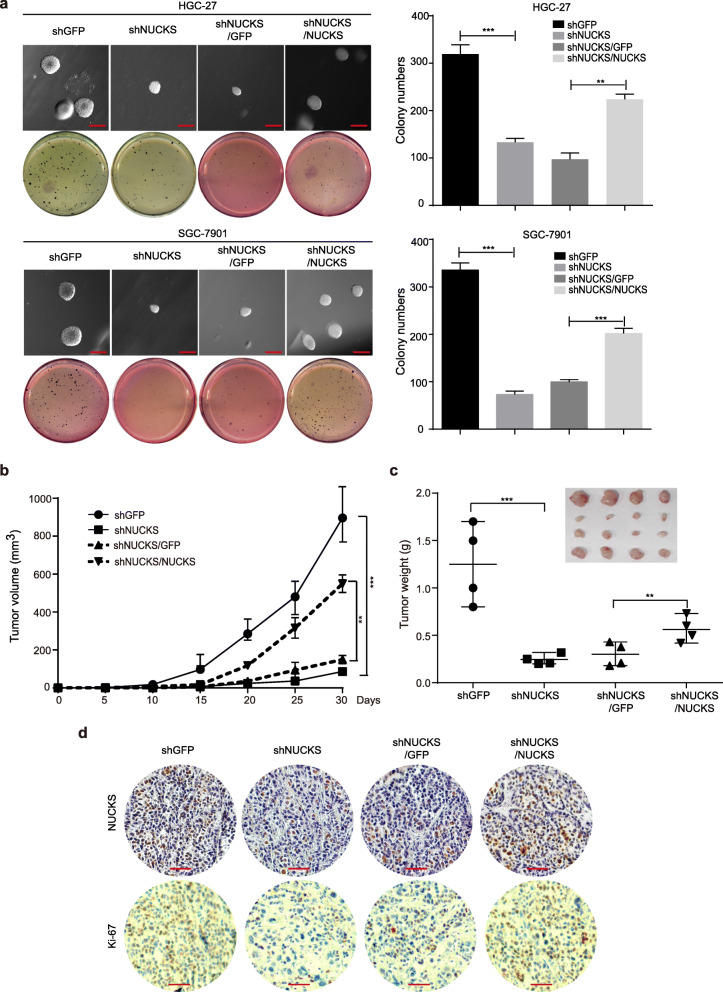
NUCKS promotes colony formation in vitro and gastric cancer cell tumor formation in vivo. **a** Soft agar assays were performed after NUCKS knockdown and restoration in the HGC-27 and SGC-7901 cell lines (Scale bars, 500 μm). The colony numbers were also quantified (**P < 0.01, ***P < 0.001). **b**, **c** Growth monitor (left), weights and photographs (right) of the indicated xenograft tumors. Data were analyzed using two-tailed Student’s t-tests (*P < 0.05, **P < 0.01, ***P < 0.001). **d** IHC analysis of the NUCKS or Ki-67 expression in the indicated xenograft tumors (Scale bars, 50 μm)

Subsequently, we examined the clonogenic abilities of the two cell lines after NUCKS knockdown using soft agar assays in vitro. As shown in Fig. 6a, smaller and fewer colonies were observed for the NUCKS knockdown cells compared with that observed in the controls. However, larger and greater numbers of colonies were observed for the NUCKS-overexpressed cells, compared with that observed using the control cells (Fig. 6a). Subcutaneous xenograft experiments with NOD/SCID mice were carried out and the results showed that the tumors formed by the NUCKS-knockdown SGC-7901 cells grew much slower, while those formed by the NUCKS-overexpressed SGC-7901 cells grew much faster (Fig. 6b). At the termination of the experiment, the mice were sacrificed, and the tumors were excised. The weights of tumors formed by NUCKS-knockdown cells were lower than those observed in the control group, while those formed by the NUCKS-overexpressed SGC-7901 cells were higher (Fig. 6c). These results indicated that NUCKS can promote the tumor growth of gastric cancer cells. To determine whether NUCKS enhances the tumor progression of gastric cancer cells by promoting cell proliferation, NUCKS and Ki-67 expression were examined in the tumor xenografts tissues by IHC analysis. As shown in Fig. 6d, the NUCKS and Ki-67 expression in the tumor tissues formed by the NUCKS-knockdown SGC-7901 cells was decreased compared with that observed in the control cells. In contrast, NUCKS and Ki-67 levels were also rescued in the tumor samples in the NUCKS-overexpressed SGC-7901 cells (Fig. 6d). Taken together, these results indicated that NUCKS most likely enhanced the tumor progression of gastric cancer cells by promoting cell growth and proliferation.

## Discussion

Several groups have reported that NUCKS is highly expressed in different human cancer cells such as breast, ovarian, lung, bone marrow and brain cancer cells, through different analyses, indicating that NUCKS may be a potential biomarker for cancer [30–32]. NUCKS overexpression was also detected in gastric adenocarcinoma by immunohistochemical analysis, and it may act as an independent prognostic factor together with Ki-67 for poor disease-free and overall survival in advanced gastric adenocarcinoma [33]. Furthermore, we observed that age, depth of invasion, lymph node metastasis, Lauren’s histological type and NUCKS expression were significantly interrelated with overall survival. There was no gender gap between NUCKS expression and overall survival. However, recent findings from the Global Burden of Diseases, Injuries, and Risk Factors Study (GBD) 2017, showed that the incidence of gastric cancer and associated death is significantly different between males and females. For males, both the age-standardized incidence and death rates of stomach cancer were more than twice the rates for females [34]. The reasons for discordance may be that more females than males (263:144) were evaluated and that the samples size of this study was small. Taken together, our findings suggest that high levels of NUCKS may be an indicator of poor prognosis in patients with gastric cancer. Using loss-of-function and gain-of-function approaches, we found that NUCKS knockdown in gastric cancer inhibited cell proliferation, self-renewal, and tumorigenesis, which could be subsequently rescued by overexpressing NUCKS. These findings reveal a novel role of NUCKS in regulating gastric cancer cell proliferation and tumorigenesis, and suggest the potential therapeutic significance of NUCKS.

Autophagy and apoptosis both play crucial roles in various biological responses [35–37]. Our findings showed that NUCKS knockdown could induce both autophagy and apoptosis. NUCKS involves in the induction of cell apoptosis in various human cancers, such as lung cancer [38], gastric cancer [29], pancreatic cancer [17]. However, the roles of NUCKS in autophagy has not been reported. We focused on studying the mechanism of NUCKS in the control of autophagy, because Huang et al. have shown that NUCKS regulates cell proliferation and apoptosis though IGF-1R and the PI3K/AKT/mTOR signaling pathway in the same gastric cancer cells [29]. Autophagy is a double-edged sword in cancer, as it can either promote or limit cancer cell survival under different conditions [39–41]. The role of autophagy in gastric cancer is very controversial. Prolonged or heightened induction of autophagy can cause autophagic cell death or type II programmed cell death, while moderate induction of autophagy is key for cell survival [42, 43]. In gastric cancer, autophagy has pro-death or pro-survival role depending on the tumor stage and cellular context [43–48]. Growing evidence suggests that autophagy can inhibit tumor growth during the early stages of tumorigenesis but promote tumor cell survival during cancer progression [42, 49–54]. Thus, the switching mechanism associated with the double-edged sword of autophagy in gastric cancer needs further in-depth research.

NUCKS has recently been reported as a positive transcriptional regulator of insulin signaling and can bind the promoter regions of mTOR [8]. In the present study, we observed that NUCKS is an autophagy regulator in gastric cancer cells. We demonstrated that NUCKS reduces autophagy via the mTOR-Beclin1 signaling pathway. The downregulation of NUCKS significantly upregulated Beclin1 levels and downregulated the phosphorylated levels of mTOR (Ser-2448) and total mTOR levels, then induced autophagy happened. Furthermore, beclin1 knockdown after NUCKS silencing markedly reduced autophagy, while using rapamycin to inhibit mTOR in NUCKS-overexpressed cells also reversed the effect of the NUCKS overexpression-mediated rescue of mTOR and LC3-II. These results demonstrated that the mTOR-Beclin1 pathway is involved in the regulation of autophagy in gastric cancer consistent with the findings of our previous study [23, 55]. However, further investigation is needed to elucidate the underlying mechanism associated with NUCKS and mTOR.

In summary, in the present study, we present evidence showing that NUCKS functions as an oncogene and an inhibitor of autophagy in gastric cancer. Thus, the downregulation or inhibition of NUCKS may be a potential therapeutic strategy for gastric cancer.

## Supplementary information

**Additional file 1: Figure S1.** (a) Kaplan-Meier analysis of progression-free survival and the log-rank test *P* values are indicated for the TCGA dataset (TGCA samples-450). (b) Kaplan-Meier analysis of progression-free survival and the log-rank test *P* values are indicated for the OncoLnc dataset (OncoLnc STAD samples-378). **Table S1.** Target Sequence for NUCKS. **Table S2.** The qRT-PCR primers.

## Data Availability

All of the data and material in this paper are available when requested.

## Abbreviations

ATCC: American Type Culture collection
Bcl-2: B-cell lymphoma-2
BrdU: 5-bromo-2′-deoxyuridine
CCNE: Cyclin E
CDKs: Cyclin-dependent kinases
CQ: Chloroquine
DAB: 3,3′-diaminobenzidine
DAPI: 4′,6-diamidino-2-phenylindole
DMEM: Dulbecco’s modified Eagle’s medium
DMSO: Dimethyl sulfoxide
FBS: Fetal bovine serum
GSEA: Gene set enrichment analysis
H&E: Hematoxylin and eosin
HMG: High mobility group
LC3B: The microtubule-associated protein light chain 3 beta
NOM: Nominal
FDR: False discovery rate
MTT: 3-(4,5-Dimethylthiazol-2-l)-2,5-Diphenyltetrazolium Bromide
mTOR: The mammalian target of rapamycin
NOD/SCID: Nonobese diabetic/severe combined immunodeficiency
NUCKS: Nuclear casein kinase and cyclin-dependent kinase substrate
P/S: Penicillin and streptomycin
qRT–PCR: The quantitative reverse transcription–PCR
shRNA: The short hairpin RNA
SPF: Specific pathogen-free
TCGA: The Cancer Genome Atlas

## References

[1] Chen W, Sun K, Zheng R, Zeng H, Zhang S, Xia C, Yang Z, Li H, Zou X, He J (2018). Cancer incidence and mortality in China, 2014. Chinese J Cancer Res.

[2] Bray F, Ferlay J, Soerjomataram I, Siegel RL, Torre LA, Jemal A (2018). Global cancer statistics 2018: GLOBOCAN estimates of incidence and mortality worldwide for 36 cancers in 185 countries. CA Cancer J Clin.

[3] Patru CL, Surlin V, Georgescu I, Patru E (2013). Current issues in gastric cancer epidemiology. Rev Med Chir Soc Med Nat Iasi.

[4] Ostvold AC, Norum JH, Mathiesen S, Wanvik B, Sefland I, Grundt K (2001). Molecular cloning of a mammalian nuclear phosphoprotein NUCKS, which serves as a substrate for Cdk1 in vivo. Eur J Biochem.

[5] Ostvold AC, Holtlund J, Laland SG (1985). A novel, highly phosphorylated protein, of the high-mobility group type, present in a variety of proliferating and non-proliferating mammalian cells. Eur J Biochem.

[6] Grundt K, Haga IV, Aleporou-Marinou V, Drosos Y, Wanvik B, Ostvold AC (2004). Characterisation of the NUCKS gene on human chromosome 1q32.1 and the presence of a homologous gene in different species. Biochem Biophys Res Commun.

[7] Grundt K, Haga IV, Huitfeldt HS, Ostvold AC (2007). Identification and characterization of two putative nuclear localization signals (NLS) in the DNA-binding protein NUCKS. Biochim Biophys Acta.

[8] Qiu B, Shi X, Wong ET, Lim J, Bezzi M, Low D, Zhou Q, Akincilar SC, Lakshmanan M, Swa HL (2014). NUCKS is a positive transcriptional regulator of insulin signaling. Cell Rep.

[9] Parplys AC, Zhao W, Sharma N, Groesser T, Liang F, Maranon DG, Leung SG, Grundt K, Dray E, Idate R (2015). NUCKS1 is a novel RAD51AP1 paralog important for homologous recombination and genome stability. Nucleic Acids Res.

[10] Kim HY, Choi BS, Kim SS, Roh TY, Park J, Yoon CH (2014). NUCKS1, a novel tat coactivator, plays a crucial role in HIV-1 replication by increasing tat-mediated viral transcription on the HIV-1 LTR promoter. Retrovirology.

[11] Yuan X, Zhang M, Ao J, Zhen Z, Gao X, Li M. NUCKS1 is a novel regulator of milk synthesis in and proliferation of mammary epithelial cells via the mTOR signaling pathway. J Cell Physiol. 2019;234(9):15825–35. 10.1002/jcp.28240 30710349

[12] Sargent LM, Ensell MX, Ostvold AC, Baldwin KT, Kashon ML, Lowry DT, Senft JR, Jefferson AM, Johnson RC, Li Z (2008). Chromosomal changes in high- and low-invasive mouse lung adenocarcinoma cell strains derived from early passage mouse lung adenocarcinoma cell strains. Toxicol Appl Pharmacol.

[13] Drosos Y, Kouloukoussa M, Ostvold AC, Grundt K, Goutas N, Vlachodimitropoulos D, Havaki S, Kollia P, Kittas C, Marinos E (2009). NUCKS overexpression in breast cancer. Cancer Cell Int.

[14] Symonowicz K, Dus-Szachniewicz K, Wozniak M, Murawski M, Kolodziej P, Osiecka B, Jurczyszyn K, Ziolkowski P (2014). Immunohistochemical study of nuclear ubiquitous casein and cyclin-dependent kinase substrate 1 in invasive breast carcinoma of no special type. Exp Ther Med.

[15] Cheong JY, Kim YB, Woo JH, Kim DK, Yeo M, Yang SJ, Yang KS, Soon SK, Wang HJ, Kim BW (2016). Identification of NUCKS1 as a putative oncogene and immunodiagnostic marker of hepatocellular carcinoma. Gene.

[16] Kikuchi A, Ishikawa T, Mogushi K, Ishiguro M, Iida S, Mizushima H, Uetake H, Tanaka H, Sugihara K (2013). Identification of NUCKS1 as a colorectal cancer prognostic marker through integrated expression and copy number analysis. Int J Cancer.

[17] Zhu K, Zhang Z, Zhang H, Wang Z, Wang F (2020). MiR-142-3p targeting NUCKS1 inhibits proliferation and invasion of pancreatic cancer cells. Artif Cells Nanomed Biotechnol.

[18] Boya P, Reggiori F, Codogno P (2013). Emerging regulation and functions of autophagy. Nat Cell Biol.

[19] Levine B (2005). Eating oneself and uninvited guests: autophagy-related pathways in cellular defense. Cell.

[20] Shimobayashi M, Hall MN (2014). Making new contacts: the mTOR network in metabolism and signalling crosstalk. Nat Rev Mol Cell Biol.

[21] Bar-Peled L, Sabatini DM (2014). Regulation of mTORC1 by amino acids. Trends Cell Biol.

[22] Laplante M, Sabatini DM (2012). mTOR signaling in growth control and disease. Cell.

[23] Zhao E, Tang C, Jiang X, Weng X, Zhong X, Zhang D, Hou J, Wang F, Huang M, Cui H (2017). Inhibition of cell proliferation and induction of autophagy by KDM2B/FBXL10 knockdown in gastric cancer cells. Cell Signal.

[24] Kim YC, Guan KL (2015). mTOR: a pharmacologic target for autophagy regulation. J Clin Invest.

[25] Wan Q, Chen H, Xiong G, Jiao R, Liu Y, Li X, Sun Y, Wang J, Yan L (2019). Artesunate protects against surgery-induced knee arthrofibrosis by activating Beclin-1-mediated autophagy via inhibition of mTOR signaling. Eur J Pharmacol.

[26] Pu Z, Wu L, Guo Y, Li G, Xiang M, Liu L, Zhan H, Zhou X, Tan H (2019). LncRNA MEG3 contributes to adenosine-induced cytotoxicity in hepatoma HepG2 cells by downregulated ILF3 and autophagy inhibition via regulation PI3K-AKT-mTOR and beclin-1 signaling pathway. J Cell Biochem.

[27] Zhao YZ, He J, Li YS, Lv SQ, Cui HJ (2020). NUSAP1 potentiates chemoresistance in glioblastoma through its SAP domain to stabilize ATR. Signal Transduct Tar.

[28] Hu J, Zhang Y, Jiang X, Zhang H, Gao Z, Li Y, Fu R, Li L, Li J, Cui H (2019). ROS-mediated activation and mitochondrial translocation of CaMKII contributes to Drp1-dependent mitochondrial fission and apoptosis in triple-negative breast cancer cells by isorhamnetin and chloroquine. J Exp Clin Cancer Res.

[29] Huang YK, Kang WM, Ma ZQ, Liu YQ, Zhou L, Yu JC (2019). NUCKS1 promotes gastric cancer cell aggressiveness by upregulating IGF-1R and subsequently activating the PI3K/Akt/mTOR signaling pathway. Carcinogenesis.

[30] Naylor TL, Greshock J, Wang Y, Colligon T, Yu QC, Clemmer V, Zaks TZ, Weber BL (2005). High resolution genomic analysis of sporadic breast cancer using array-based comparative genomic hybridization. Breast cancer research : BCR.

[31] Thompson HG, Harris JW, Wold BJ, Quake SR, Brody JP (2002). Identification and confirmation of a module of coexpressed genes. Genome Res.

[32] Schaner ME, Ross DT, Ciaravino G, Sorlie T, Troyanskaya O, Diehn M, Wang YC, Duran GE, Sikic TL, Caldeira S (2003). Gene expression patterns in ovarian carcinomas. Mol Biol Cell.

[33] Yang M, Wang X, Zhao Q, Liu T, Yao G, Chen W, Li Z, Huang X, Zhang Y (2014). Combined evaluation of the expression of NUCKS and Ki-67 proteins as independent prognostic factors for patients with gastric adenocarcinoma. Tumour Biol.

[34] Collaborators GBDSC (2020). The global, regional, and national burden of stomach cancer in 195 countries, 1990-2017: a systematic analysis for the global burden of disease study 2017. Lancet Gastroenterol Hepatol.

[35] Zhao E, Hou J, Ke X, Abbas MN, Kausar S, Zhang L, Cui H (2019). The Roles of Sirtuin Family Proteins in Cancer Progression. Cancers (Basel).

[36] Fitzwalter BE, Towers CG, Sullivan KD, Andrysik Z, Hoh M, Ludwig M, O'Prey J, Ryan KM, Espinosa JM, Morgan MJ (2018). Autophagy Inhibition Mediates Apoptosis Sensitization in Cancer Therapy by Relieving FOXO3a Turnover. Developmental Cell.

[37] Fitzwalter BE, Thorburn A (2018). FOXO3 links autophagy to apoptosis. Autophagy.

[38] Shen H, Wang L, Ge X, Jiang CF, Shi ZM, Li DM, Liu WT, Yu XB, Shu YQ (2016). MicroRNA-137 inhibits tumor growth and sensitizes chemosensitivity to paclitaxel and cisplatin in lung cancer. Oncotarget.

[39] Xuan F, Huang M, Liu W, Ding H, Yang L, Cui H (2016). Homeobox C9 suppresses Beclin1-mediated autophagy in glioblastoma by directly inhibiting the transcription of death-associated protein kinase 1. Neuro-oncology.

[40] White E (2012). Deconvoluting the context-dependent role for autophagy in cancer. Nat Rev Cancer.

[41] Brech A, Ahlquist T, Lothe RA, Stenmark H (2009). Autophagy in tumour suppression and promotion. Mol Oncol.

[42] Qiu GL, Li XQ, Che XM, Wei C, He SC, Lu J, Jia ZL, Pang K, Fan L (2015). SIRT1 is a regulator of autophagy: implications in gastric cancer progression and treatment. FEBS Lett.

[43] Codogno P, Meijer AJ (2005). Autophagy and signaling: their role in cell survival and cell death. Cell Death Differ.

[44] Zhang YJ, Zhang L, Gao JH, Wen LP (2019). Pro-death or pro-survival: contrasting paradigms on nanomaterial-induced autophagy and exploitations for Cancer therapy. Accounts Chem Res.

[45] Yang ZNJ, Chee CE, Huang SB, Sinicrope FA (2011). The role of autophagy in Cancer: therapeutic implications. Mol Cancer Ther.

[46] Hwang B, Lee S, Jung C, Yu B, Lee Y. Regulation and functional roles of autophagy in Helicobacter pylori CagA-mediated gastric cancer. Cancer Res. 2019;79(13):2.

[47] Hwang BR, Lee SD, Lee YC (2018). Functional role of helicobacter pylori Cytotoxin-associated gene a(Caga) induced autophagy in gastric Cancer cells. Gastroenterology.

[48] Qian HR, Yang Y (2016). Functional role of autophagy in gastric cancer. Oncotarget.

[49] Rouschop KMA, Wouters BG (2009). Regulation of autophagy through multiple independent hypoxic signaling pathways. Curr Mol Med.

[50] Chen N, Debnath J (2010). Autophagy and tumorigenesis. FEBS Lett.

[51] Degenhardt K, Mathew R, Beaudoin B, Bray K, Anderson D, Chen GH, Mukherjee C, Shi YF, Gelinas C, Fan YJ (2006). Autophagy promotes tumor cell survival and restricts necrosis, inflammation, and tumorigenesis. Cancer Cell.

[52] Amaravadi R, Kimmelman AC, White E (2016). Recent insights into the function of autophagy in cancer. Genes Dev.

[53] Zhou HY, Yuan M, Yu QF, Zhou XY, Min WP, Gao D (2016). Autophagy regulation and its role in gastric cancer and colorectal cancer. Cancer Biomarkers.

[54] Cao YJ, Luo YC, Zou J, Ouyang J, Cai ZH, Zeng X, Ling H, Zeng TB (2019). Autophagy and its role in gastric cancer. Clin Chim Acta.

[55] Liu JZ, Hu YL, Feng Y, Jiang Y, Guo YB, Liu YF, Chen X, Yang JL, Chen YY, Mao QS (2020). BDH2 triggers ROS-induced cell death and autophagy by promoting Nrf2 ubiquitination in gastric cancer. J Exp Clin Canc Res.

