# Supplementary material for: NUCKS promotes cell proliferation and suppresses autophagy through the mTOR-Beclin1 pathway in gastric cancer
Source: J Exp Clin Cancer Res. 2020 Sep 21;39:194. doi: 10.1186/s13046-020-01696-7 (PMC7504682; doi:10.1186/s13046-020-01696-7)
Supplement: Supplementary file 1 — Additional file 1: Figure S1. (a) Kaplan-Meier analysis of progression-free survival and the log-rank test P values are indicated for the TCGA dataset (TGCA samples-450). (b) Kaplan-Meier analysis of progression-free survival and the log-rank test P values are indicated for the OncoLnc dataset (OncoLnc STAD samples-378). Table S1. Target Sequence for NUCKS. Table S2. The qRT-PCR primers. [file 13046_2020_1696_MOESM1_ESM.docx]

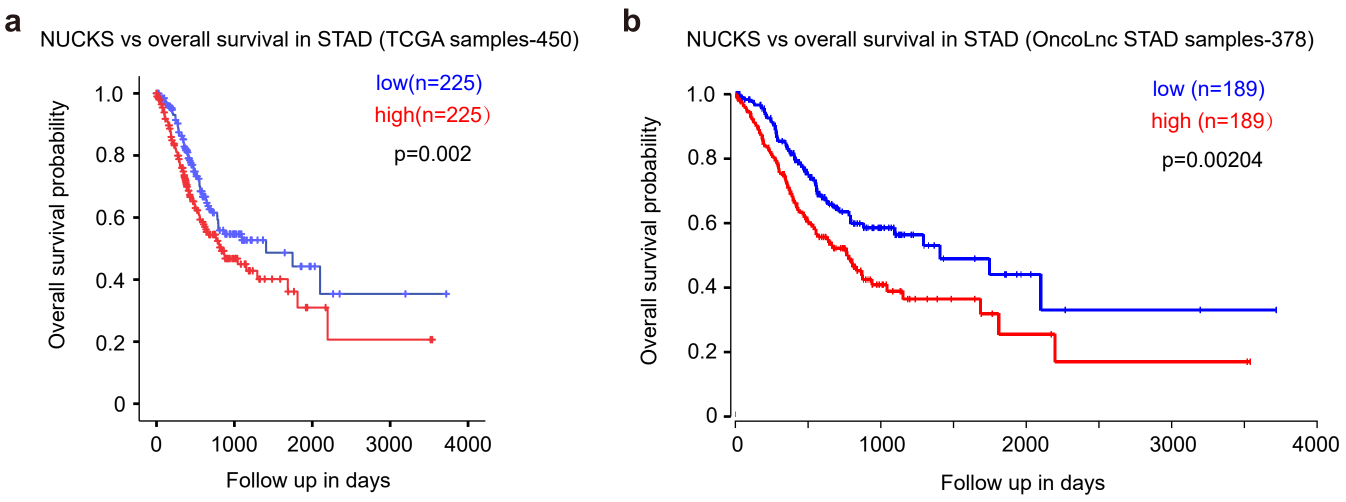
a
NUCKS vs overall survival in STAD (TCGA samples-450)
low(n=225)
high(n=225)
p=0.002
Overall survival probability
1.0
0.8
0.6
0.4
0.2
0
0
1000
2000
3000
4000
Follow up in days
b
NUCKS vs overall survival in STAD (OncoLnc STAD samples-378)
low (n=189)
high (n=189)
p=0.00204
Overall survival probability
1.0
0.8
0.6
0.4
0.2
0
0
1000
2000
3000
4000
Follow up in days
